# ﻿A new chemosymbiotic bivalve species of the genus *Acharax* Dall, 1908 (Bivalvia, Solemyida, Solemyidae) from the Haima cold seep of the South China Sea

**DOI:** 10.3897/zookeys.1198.112618

**Published:** 2024-04-24

**Authors:** Mei Yang, Baoquan Li, Zhibin Gan, Dong Dong, Xinzheng Li

**Affiliations:** 1 Department of Marine Organism Taxonomy & Phylogeny, Institute of Oceanology, Chinese Academy of Sciences, Qingdao 266071, China Institute of Oceanology, Chinese Academy of Sciences Qingdao China; 2 Yantai Institute of Coastal Zone Research, Chinese Academy of Sciences, Yantai 264003, China Yantai Institute of Coastal Zone Research, Chinese Academy of Sciences Yantai China; 3 Center for Ocean Mega-Science, Chinese Academy of Sciences, Qingdao 266071, China Center for Ocean Mega-Science, Chinese Academy of Sciences Qingdao China; 4 University of Chinese Academy of Sciences, Beijing 100049, China University of Chinese Academy of Sciences Beijing China

**Keywords:** *Acharaxhaimaensis* sp. nov., Bathyal, deep-sea, taxonomy

## Abstract

Solemyidae is an ancient group of protobranch bivalves that typically inhabit unusual environments, such as deep-sea chemosynthetic environments, and are symbiotic with chemoautotrophic and gill-hosted bacteria. In May 2018, a living solemyid bivalve was collected using a remotely operated vehicle at a depth of 1,390 m from the Haima cold seep in the northwestern slope of the South China Sea. Through a comprehensive taxonomic approach combining morphological observations and molecular phylogeny reconstruction of concatenated mitochondrial COI,16S rRNA and 18S rRNA gene sequences, a new species, *Acharaxhaimaensis***sp. nov.** is identified and described. The discovery of this new species contributes to the diversity of known solemyids in deep-sea chemosynthetic environments.

## ﻿Introduction

Solemyidae is a group of ancestral protobranch bivalves (Pojeta 1988) with a worldwide marine distribution across various depths ranging from 0 to 6,000 m (Conway et al. 1992; Fujiwara et al. 2003). This family consists of over 30 valid living species that are classified into two reciprocally monophyletic genera: *Acharax* Dall, 1908 and *Solemyas.l.* Gray 1840. These genera are distinguished by their unique thickened frill of radially pleated periostracum, which extends beyond the calcified shell margins. The main difference between these genera lies in the position of the ligament; it is external in *Acharax* and internal in *Solemyas.l.* (Taylor et al. 2008; Kamenev 2009; Oliver et al. 2011; Sharma et al. 2013). Solemyid bivalves predominantly inhabit chemosynthesis-based ecosystems (Conway et al. 1992; Walton 2015; Bailey 2021) and rely on intracellular chemosynthetic symbionts for nutrition (Fisher and Childress 1986; Rodrigues et al. 2010; Fukasawa et al. 2017).

The classification of Solemyidae based on external features is problematic (Kamenev 2009; Oliver et al. 2011; Bailey 2021) due to the lack of distinguishing characteristics such as shell sculpture and hinge teeth. These bivalves are uniformly covered by a distinctive, thick, shiny periostracum (Taylor et al. 2008). Furthermore, there has been limited research on the morphology and molecular aspects of solemyid bivalves. As a result, the taxonomy and systematic status of Solemyidae have remained problematic. Recently, Sato et al. (2013) described and depicted the shell microstructures of five solemyid species from Japan by scanning electron microscopy.

In this study, we diagnose and describe a new *Acharax* species from the Haima cold seep, China. Additionally, we conducted an analysis of interspecific genetic distances within the family Solemyidae based on the mitochondrial cytochrome *c* oxidase subunit I (COI) gene. Furthermore, we examined the phylogenetic relationships within the order Solemyida using COI,16S rRNA and 18S rRNAgene sequences.

## ﻿Materials and methods

**Figure 1.**
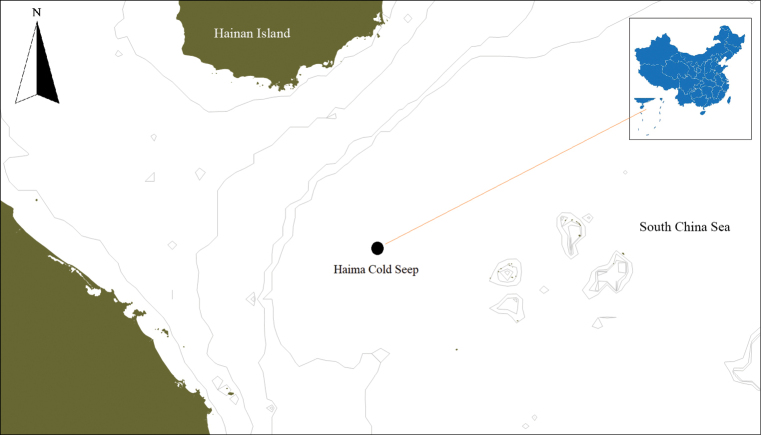
Sampling site of *Acharaxhaimaensis* sp. nov.

The specimen was collected from the Haima cold seep in the northwestern slope of the South China Sea at a depth of 1,390 m using a remotely operated vehicle (ROV) in 2018 (Fig. 1). On board, the specimen was photographed with a Canon EOS-1D digital single lens reflex camera. Then, the specimen was fixed in 95% ethanol and deposited in the Marine Biological Museum of the Chinese Academy of Sciences (**MBMCAS**) in the Institute of Oceanology, Chinese Academy of Sciences, Qingdao. Measurements were taken point-to-point with digital calipers, recorded to the nearest 0.1 mm.

Total genomic DNA was extracted from the muscle tissues using TIANamp Marine Animals DNA Kit (TIANGEN, China) following the manufacturer’s instructions. Then the genomic DNA was used for Illumina sequencing and Oxford Nanopore sequencing (Shanghai BIOZERON Co. Ltd). After assembly and annotation, we successfully obtained the complete mitochondrial genome of the new *Acharax* specimen (GenBank accession number: ON023263).

The Kimura’s 2-parameter (K2P) genetic distances between COI sequences of solemyid species were estimated by MEGA 6.06 (Tamura et al. 2013). The phylogenetic relationships within Solemyida were conducted using COI, 16S rRNA and 18S rRNA gene sequences from 17 in-group species (Suppl. material 1). The nucleotide sequences of each gene were aligned in batches using MAFFT (Katoh et al. 2019), and ambiguously aligned regions were deleted using Gblocks 0.91b (Gblocks parameters: minimum length of a block = 5; allowed gap positions = with half) (Talavera and Castresana 2007). Subsequently, the sequences were concatenated into a single alignment used to generate nexus files in PhyloSuite 1.2.2 (Zhang et al. 2020). A maximum-likelihood analysis was performed with the GTR+I+G substitution model in IQ-TREE (Nguyen et al. 2015), and branch support was evaluated with ultrafast bootstrap (UFB) with 1,000 replicates. The phylogenetic tree and node labels were graphically edited with iTOL (Letunic and Bork 2007).

## ﻿Results

### ﻿Systematics

**Order Solemyoida Dall, 1889**

**Superfamily Solemyoidea Gray, 1840**

**Family Solemyidae Gray, 1840**

#### ﻿Genus Acharax

Taxon classificationAnimaliaSolemyidaSolemyidae

Dall, 1908

3B302A17-451D-5F28-9B0B-03CABCD9F21A

##### Type species.

*Solemyajohnsoni* Dall, 1891; Recent, North Pacific.

#### ﻿ Acharax haimaensis sp. nov.

Taxon classificationAnimaliaSolemyidaSolemyidae

B5361E45-514F-565C-809E-422EC282F7CD

https://zoobank.org/2356CE0D-13A0-4AA7-A322-D5ACD518F028

Fig. 2

##### Material examined.

***Holotype***: Complete, Haima cold seep cruise, HOV *Shen Hai Yong Shi* 71, 1390 m, coll. crew of R/V *Tan Suo Yi Hao*, 17 May 2018, MBM287872.

**Figure 2.**
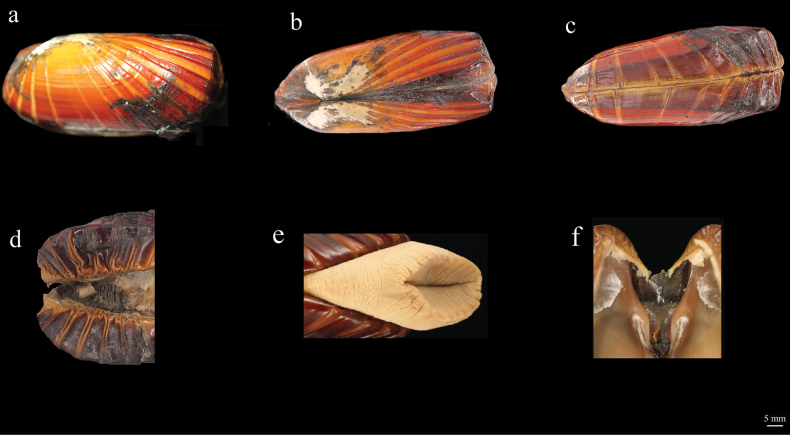
Holotype of *Acharaxhaimaensis* sp. nov.

##### Description.

***Shell***: the shell is elongate, rectangular, equivalve, and robust. Shell length 103.32 mm, width 48.68 mm, height 45.20 mm. The length/width ratio and length/height ratio are 2.12 and 2.29, respectively. The beak is positioned at approximately the posterior quarter of the shell. The anterior margin is broadly rounded and compressed medially, while the posterior margin is tightly rounded. The ventral margin is straight and shallowly concave towards the posterior. The periostracum extends well beyond the calcified shell margin, folding inwards, and reddish brown but gradually paler towards the prodissoconch. The shell has radial ridges with strong, flattened summits. The ridges are more crowded anteriorly and posteriorly, and vary in both width and colour. There are four closely spaced ridges over the posterior area, and the median area is almost smooth, with two or three low ribs. The anterior region with eight or nine deeply cut ribs. Hinge teeth are absent, and the ligament is mainly external. The posterior siphon aperture is lined by two rings of papillae. The foot is large, with a broadly oval sole, and its margin interdigitates between large and small blunt papillae.

***Adductor muscle scar and pallial scars***: the anterior adductor-muscle scar has a D-shaped, irregularly and posteriorly oriented straight face. There is a loop of muscle scar that extends from the hinge plane, reaching halfway along the valve and connecting ventrally to adductor scar. The posterior adductor scar is ovoid, with irregular contours, and exhibits faint radial sculpture. It is separated by a line that extends from the scar to the beak along a subtle depression. The foot is flattened at anterior end when open. The flattened face is longitudinally bifurcated, displaying horizontal lamellae, and is fringed with approximately 32 short, adze-shaped ridges. These ridges interlock when the foot is closed, and each ridge is topped with a small, rounded papilla. The mantle is fused along the ventral margin and covers large gills that extend about three-fifths of the total length of the body.

##### Etymology.

Named after the type locality, the Haima cold seep in the northwestern slope of the South China Sea.

##### Distribution.

Currently, *Acharaxhaimaensis* sp. nov. is known only from the type locality, in the northwestern slope of the South China Sea, at a depth of 1,390 m.

##### Remarks.

The genus *Acharax* has a worldwide distribution in cold seep habitats with sulfide present (Sibuet and Olu 1998) at depths ranging from approximately 400 m to 6,000 m (Neulinger et al. 2006). *Acharax* has approximately nine extant species (WoRMs 2024) and more than 20 fossil seep species (Amano and Ando 2011; Saether et al. 2016; Isaji and Kato 2017; Hansen et al. 2020). *Acharaxclarificata* Dell, 1995 closely resembles to our specimen, but the new species differs from *A.clarificata* in the shape of the shell. Our specimen has an elongate, nearly rectangular shell with parallel dorsal and ventral margins, whereas *A.clarificata* has a shallowly concave ventral margin and is particularly more deeply concave towards the posterior end.

The genus *Acharax* exhibits distinct morphological differences from *Solemya*, including a prominent large external ligament located on a narrow nymph and larger size (Walton 2015). However, due to the striking similarities in gross morphology with *Solemya*, there is a possibility that *Acharax* species have been misclassified as *Solemya* in the past (Sibuet and Olu 1998). Even within the genus *Acharax*, certain species share morphological similarities, but molecular data suggests the presence of cryptic speciation (Neulinger et al. 2006). Therefore, gene-sequence analysis can provide valuable information for the classification of solemyid bivalves.

##### Molecular support.

**Figure 3.**
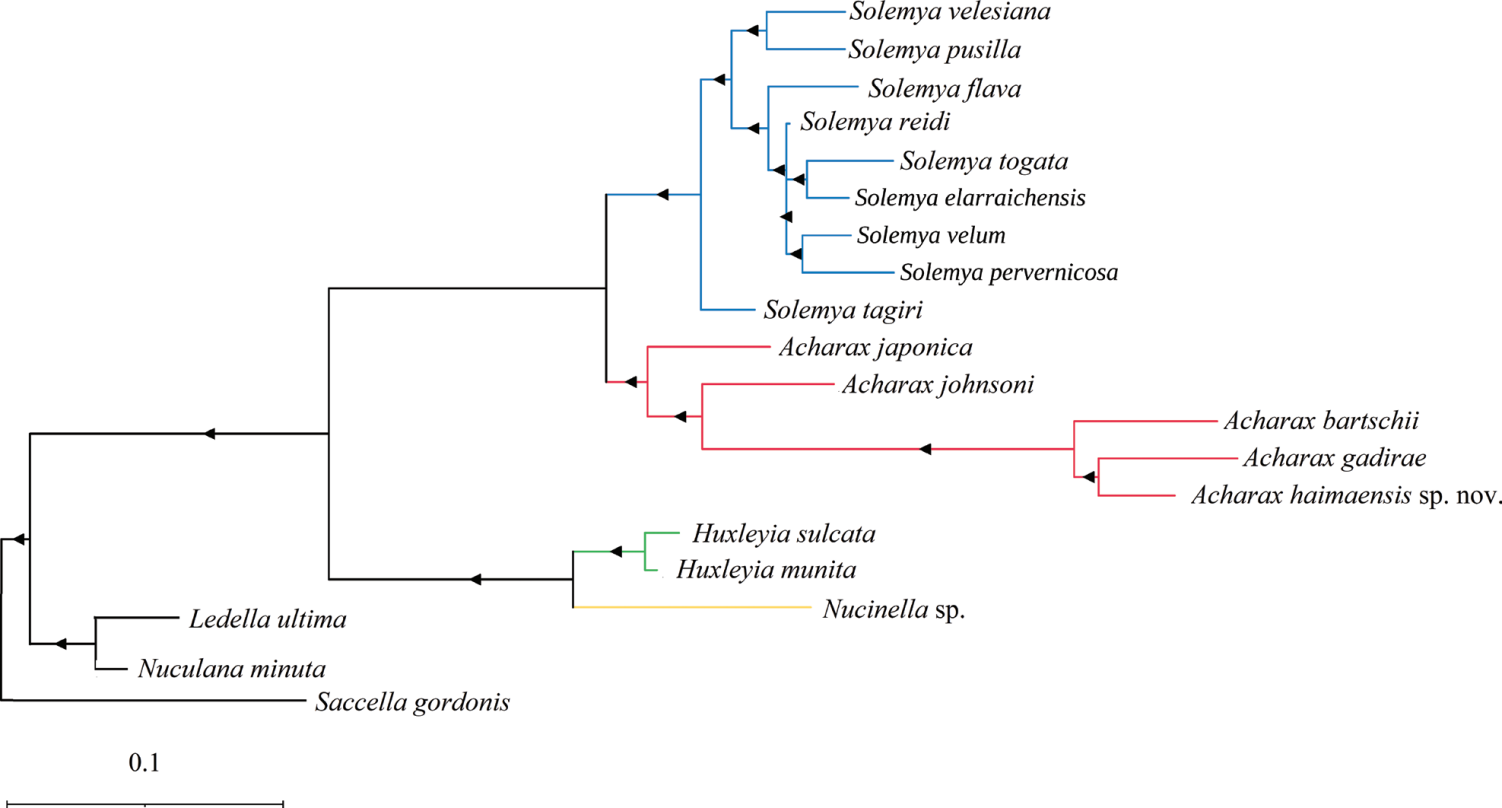
Phylogenetic relationships of Solemyida by the ML analysis of mitochondrial (COI+16S rRNA+18S rRNA) sequences. The black triangles demonstrate bootstrap values ≥95% for the node.

The genetic divergence between *Acharaxhaimaensis* sp. nov. and the solemyid species analyzed ranged from 21.4% (*A.johnsoni*) to 28.2% (*Solemyapusilla*) (Table 1). It is evident that the lowest genetic distance was observed between the two *Acharax* species. The phylogenetic tree of the family Solemyidae, reconstructed using maximum likelihood based on mitochondrial COI,16S rRNA and 18S rRNA sequence data, is shown in Fig. 3. Both *Acharax* and *Solemya* formed monophyletic clades with strong support values (≥95%).

**Table 1.** Kimura’s 2-parameter pair-wise genetic distances (in percentage) between species of Solemyidae using COI gene sequences.

Species	1	2	3	4	5	6	7	8	9
*Acharaxhaimaensis*	—								
*Acharaxjohnsoni*	21.4	—							
*Solemyaelarraichensis*	27.8	28.0	—						
*Solemyaflava*	25.8	29.5	18.4	—					
*Solemyapervernicosa*	23.4	22.6	26.4	24.5	—				
*Solemyapusilla*	28.2	25.0	17.3	20.4	27.7	—			
*Solemyatagiri*	25.1	22.7	19.8	19.6	25.1	18.8	—		
*Solemyavelesiana*	25.6	24.0	17.7	19.6	24.6	12.2	15.2	—	
*Solemyavelum*	27.5	29.6	16.4	17.8	29.1	18.7	16.5	15.6	—

The placement of the new species, *Acharaxhaimaensis* sp. nov., within the genus *Acharax* is supported by both the morphological and molecular phylogenetic analyses. Its discovery at the Haima cold seep contributes to the known diversity of solemyids in chemosynthesis-based ecosystems. The Protobranchia represent an intriguing group of Bivalvia in terms of their early evolution, unique anatomy, larval development, and ecological diversification. However, there are still controversies surrounding the origin and evolutionary process of Protobranchia, particularly the phylogenetic relationships among higher taxa. Currently, research into the morphological taxonomy and molecular systematics of the Protobranchia is relatively limited. More comprehensive taxon collections in the future will be necessary to lead us closer to the goal of reconstructing the evolutionary history of protobranch bivalves.

## Supplementary Material

XML Treatment for Acharax

XML Treatment for Acharax haimaensis

## ﻿Additional information

### Data availability

All of the data that support the findings of this study are available in the main text or Supplementary Information.

## Supplementary materials

Supplementary material 1 The mitochondrial gene sequences used in this study Data type docx https://binary.pensoft.net/file/1032132This dataset is made available under the Open Database License (http://opendatacommons.org/licenses/odbl/1.0/). The Open Database License (ODbL) is a license agreement intended to allow users to freely share, modify, and use this Dataset while maintaining this same freedom for others, provided that the original source and author(s) are credited.Mei Yang, Baoquan Li, Zhibin Gan, Dong Dong, Xinzheng Li
