# Supplementary material for: ﻿A new chemosymbiotic bivalve species of the genus Acharax Dall, 1908 (Bivalvia, Solemyida, Solemyidae) from the Haima cold seep of the South China Sea
Source: Zookeys. 2024 Apr 24;1198:185–92. doi: 10.3897/zookeys.1198.112618 (PMC11063622; doi:10.3897/zookeys.1198.112618)
Supplement: Supplementary material 1 — The mitochondrial gene sequences used in this study [file zookeys-1198-185_article-112618__-s001.docx]

**Supplementary Table S1**

The mitochondrial gene sequences used in this study.

| Species | COI | 16S rRNA | 18S rRNA |
| --- | --- | --- | --- |
| *Acharax bartschii* | - | KC984671 | KC984714 |
| *Acharax gadirae* | - | KC984672 | KC984715 |
| *Acharax* *haimaensis* sp. nov. | ON023263 | ON023263 | - |
| *Acharax japonica* | LC186990 | LC144670 | LC186954 |
| *Acharax johnsoni* | LC186991 | LC144668 | LC144714 |
| *Solemya elarraichensis* | KC984743 | KC984673 | KC984719 |
| *Solemya flava* | LC144809 | LC144672 | LC144710 |
| *Solemya pervernicosa* | LC187004 | LC144671 | LC144712 |
| *Solemya pusilla* | LC144807 | LC144673 | LC144716 |
| *Solemya reidi* | - | L07864 | AF117737 |
| *Solemya tagiri* | LC187006 | - | LC186975 |
| *Solemya togata* | AJ389658 | - | - |
| *Solemya velesiana* | KC984744 | KC984674 | KC984717 |
| *Solemya velum* | KC984745 | NC_017612 | KC984718 |
| *Huxleyia munita* | - | - | KC429323 |
| *Huxleyia sulcata* | - | - | LC144700 |
| *Nucinella* sp. | KC429089 | - | KC429324 |
| *Ledella ultima* | KC984740 | KC984667 | KC984685 |
| *Nuculana minuta* | DQ280018 | DQ280030 | DQ279938 |
| *Saccella gordonis* | LC144826 | LC144680 | LC144694 |
